# P18. The tumour leukocyte infiltrate is the key predictor for therapeutic response to catumaxomab therapy

**DOI:** 10.1186/2051-1426-2-S2-P9

**Published:** 2014-03-12

**Authors:** K Weiler, L Rava, M Jäger, I Funke, M Joka, KW Jauch, B Mayer

**Affiliations:** 1 University Hospital of Munich LMU Klinikum Großhadern, Surgery, Munich, Germany; 2 Trion Research, Munich, Germany; 3 SpheroTec GmbH, Munich, Germany

## Background

Patient stratification for therapeutic response mainly focus on biomarker expression on cancer cells. Contrary, the tumour micromileu is less considered, although stromal cells and leukocytes play a crucial role in tumour progression and drug resistance. The present study investigates the impact of tumour infiltrating lymphocytes on catumaxomab efficacy in CRC.

## Patients and methods

Fresh tumour samples from 27 CRC patients were used to prepare tumour spheroids. Autologous PBMCs were isolated by ficoll density gradient. Tumour spheroids co-cultured with or without PBMCs were treated with catumaxomab for 96h. The cellular composition of the spheroids and the therapeutic impact on epithelial cells and leukocytes were measured by FACS analysis. The metabolic activity of the co-cultures was determined by the ATP assay.

## Results

Similar to their original cancers all spheroid models consisted of a sufficient high fraction of EpCAM positive tumour cells indicating CRC an appropriate cancer type for catumaxomab therapy. In contrast, the fraction of CD45 positive leukocytes in spheroids was low mimicking the parental tumours. The effector cell to target cell (E:T, CD45 : EpCAM) ratio ranged from 0,05:1 to 1,92:1. Treatment of these spheroids with catumaxomab revealed no significant therapeutic effect. Addition of patient specific PBMCs changed the E:T ratio up to 7:1 and had a significant impact on antibody efficacy. Catumaxomab induced cell death was found up to 52% (p=0,015) depending on the individual E:T ratio. After catumaxomab treatment co-cultures demonstrated metabolic stimulation underlining the functionality of the leukocytes.

## Conclusions

A dual biomarker system is required to select appropriate CRC patients for catumaxomab treatment. EpCAM expression on cancer cells is required, but the leukocyte infiltrate is the key predictor for the therapeutic response of catumaxomab. Both extent and functionality of the tumour infiltrating leukocytes have to be determined in the individual tumour sample for targeted therapy using the trifunctional monoclonal antibody.

